# FoxM1, a Forkhead Transcription Factor Is a Master Cell Cycle Regulator for Mouse Mature T Cells but Not Double Positive Thymocytes

**DOI:** 10.1371/journal.pone.0009229

**Published:** 2010-02-16

**Authors:** Ling Xue, Leslie Chiang, Bo He, You-Yang Zhao, Astar Winoto

**Affiliations:** 1 Department of Molecular and Cell Biology and Cancer Research Laboratory, University of California at Berkeley, Berkeley, California, United States of America; 2 Department of Pharmacology and Center for Lung and Vascular Biology, University of Illinois College of Medicine, Chicago, Illinois, United States of America; New York University, United States of America

## Abstract

FoxM1 is a forkhead box transcription factor and a known master regulator required for different phases of the cell cycle. In cell lines, FoxM1 deficient cells exhibit delayed S phase entry, aneuploidy, polyploidy and can't complete mitosis. *In vivo*, FoxM1 is expressed mostly in proliferating cells but is surprisingly also found in non-proliferating CD4^+^CD8^+^ double positive thymocytes. Here, we addressed the role of FoxM1 in T cell development by generating and analyzing two different lines of T-cell specific FoxM1 deficient mice. As expected, FoxM1 is required for proliferation of early thymocytes and activated mature T cells. Defective expression of many cell cycle proteins was detected, including cyclin A, cyclin B1, cdc2, cdk2, p27 and the Rb family members p107 and p130 but surprisingly not survivin. Unexpectedly, loss of FoxM1 only affects a few cell cycle proteins in CD4^+^CD8^+^ thymocytes and has little effect on their sensitivity to apoptosis and the subsequent steps of T cell differentiation. Thus, regulation of cell cycle genes by FoxM1 is stage- and context-dependent.

## Introduction

Proliferation and apoptosis are integral parts of T cell development. Early thymocytes die if their T-cell receptor (TCR) β genes do not rearrange successfully to produce a functional protein. Formation of a cell surface pre-TCR complex, consisting of TCRβ/pTα/CD3 will result in proliferation and differentiation of CD4^−^CD8^−^ (DN) thymocytes into CD4^+^CD8^+^(DP) thymocytes [1]–[5]. DP cells are exquisitely sensitive to apoptosis and live only for 3–5 days *in vivo*. Only a small fraction of DP cells with the appropriate TCRαβ/CD3 complex can differentiate into CD4^+^CD8^−^ or CD4^−^CD8^+^ SP thymocytes. These cells migrate to the peripheral immune organs, spleen and lymph nodes. Early in life, when these organs are relatively empty, homeostatic proliferation of SP cells takes place to fill the empty niches. This homeostatic proliferation ceases in adult mice. Mature T cells exist in the G_0_ state of the cell cycle and proliferate only when stimulated through their TCR complex.

Regardless of their cell types, cell cycle progression in mammalian cells is tightly regulated by the rise and fall of the cyclin/CDK kinase activities. Entry into the S phase requires activation of the CDK4/cyclin D or CDK6/cyclin D and the CDK2/cyclin E activities [6], [7]. The latter kinase phosphorylates the Rb family members Rb, p107 and p130, which then release the E2F transcription factors required for cell entry into the S phase [8], [9]. In S phase, CDK2/cyclin A is active while cyclin B/CDK1 is crucial for the G_2_/M phases of the cell cycle. FoxM1 belongs to the forkhead family of transcription factors that include Foxp3, Foxo3 and other Fox family proteins crucial for a wide variety of biological processes [10]–[15]. FoxM1 is known to be a master regulator of cell cycle proteins that are expressed in proliferating cells. Its known direct target genes include cyclin D1, c-myc, cyclin B1, p27, p21, G_2_/M specific protein survivin and many other cell cycle related proteins. In cell lines and some primary cells, FoxM1 deficient cells fail to proliferate and exhibit aneuploidy and polyploidy [15], [16] with many similarities to that of survivin deficient cells [17], [18]. In adult mice, FoxM1 is highly expressed in the testis, small intestine, colon and thymus [19], [20]. Thymus contains different T-cell populations but only a small fraction is proliferating (DN thymocytes). FoxM1 presumably is expressed in proliferating DN thymocytes but it is also found in DP cells, which constitute >85% thymocytes. However, DP thymocytes exist in 2N DNA content and do not proliferate [21]. In addition to FoxM1, DP thymocytes express many cell cycle proteins, including survivin, cyclin A, cyclin B1, cyclin D, CDK2, cdc2, p27, p21 and the RB family members p107, p130. Differentiation of DP thymocytes into SP cells results in down-regulation of FoxM1 as well as other cell cycle proteins [21], [22]. We hypothesized that expression of these cell cycle genes in DP thymocytes might contribute to their inherent sensitivity to apoptosis. As FoxM1 is a master cell cycle gene for many cell cycle proteins, we deleted FoxM1 from DP thymocytes by crossing floxed FoxM1 mice [23] to two different T cell specific Cre transgenic mice (*lck-Cre* and *CD4-Cre*) [24], [25]. Analysis of *lck-Cre/FoxM1^fl/fl^* mice showed that deletion of FoxM1 starting from DN thymocytes results in a 2-fold drop of the DP thymocyte population followed by a similar drop of mature peripheral T cells. In contrast, T cell development appears to be normal in *CD4-Cre/FoxM1^fl/fl^* mice although mature T cells exhibit defective proliferation when stimulated. Interestingly, expression of a few cell cycle proteins is affected in FoxM1-deficient DP thymocytes. Apoptosis of FoxM1-deficient DP cells appears to be normal. We concluded that FoxM1 is an important factor for mature T cells and early T cell subsets but not for DP thymocytes.

## Results

### Abnormal T Cell Development of *lck-Cre/FoxM1^fl/fl^* Mice

**Figure 1:**
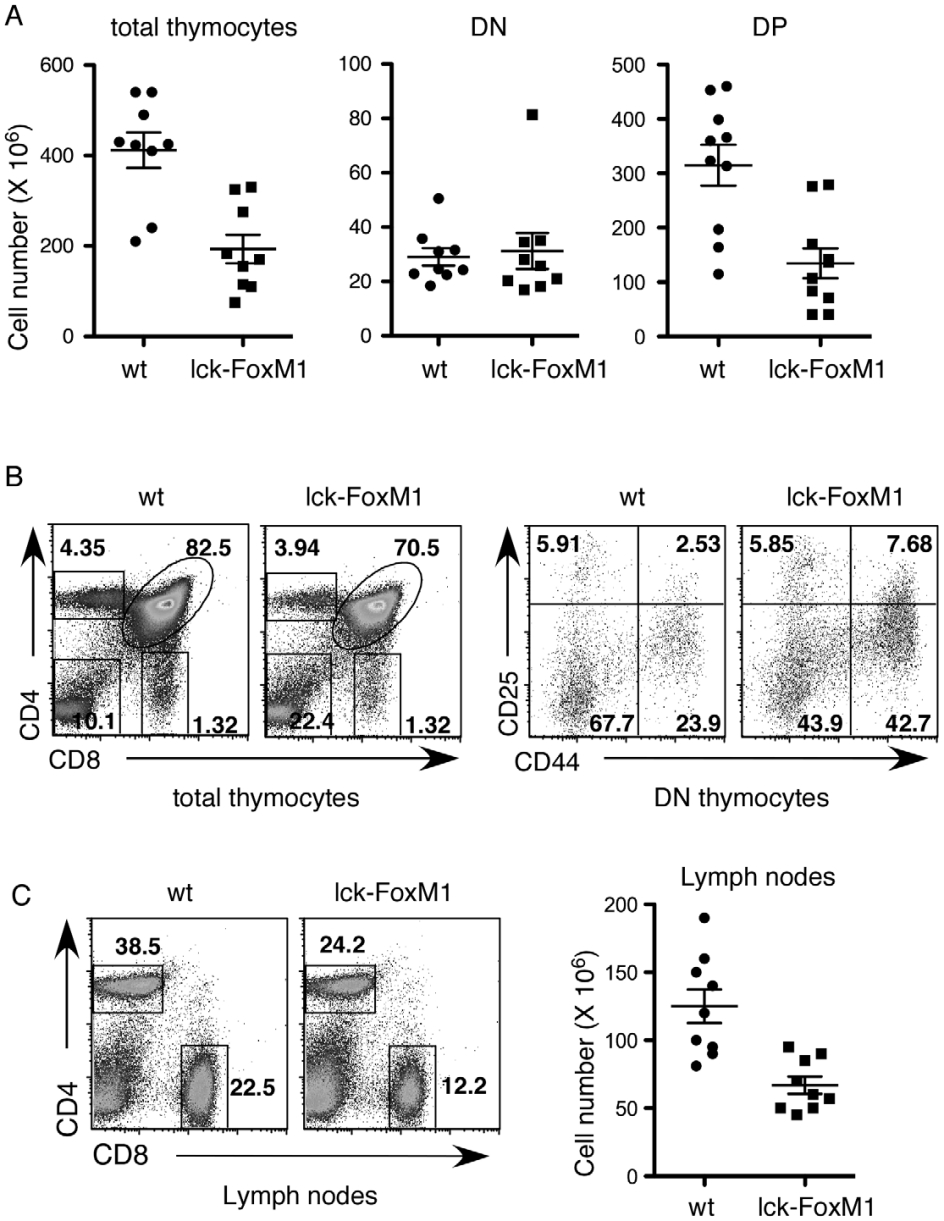
Characterization of lck-FoxM1 mice. (A) The total number of thymocytes, double negative (DN) cells, and double positive (DP) cells from each mouse are depicted as a box or a circle (*n* = 9). The average number of the total thymocytes (X 10^6^) are 412±117 for wild-type mice and 193±95 for the lck-FoxM1 mice (p<0.05). The average number (X 10^6^) for DN thymocytes are 29.1±9.7 for wild-type mice and 31.2±19.9 for lck-FoxM1 mice. For DP thymocytes, the average number (X 10^6^) is 315±119 for wild-type mice and 134±86 for lck-FoxM1 mice (p<0.05). (B) Representative plots of CD4 *versus* CD8 profiles of total thymocytes and CD44 *versus* CD25 profiles of gated DN thymocytes are shown. (C) CD4 *versus* CD8 profiles and the total number of cells in lymph nodes are shown here. Right panel: Each box or circle represents one mouse (*n* = 9). The average number of the total lymph nodes cells (X 10^6^) are 125±37 for wild-type mice and 66.9±18.9 for the lck-FoxM1 mice (p<0.05).

We crossed floxed *FoxM1* mice [23], [26] to *lck-Cre* mice to generate *lck-Cre/FoxM1^fl/fl^* mice (henceforth termed lck-FoxM1). In these mice, Cre is expressed early during development starting in the DN2 T cell population (CD4^−^CD8^−^ CD25^+^ CD44^+^) [18]. Deletion of FoxM1 is thus expected to occur before the pre-TCR mediated proliferation that takes place between DN3 (CD4^−^CD8^−^ CD25^−^CD44^+^) and DN4 (CD4^−^CD8^−^ CD25^−^CD44^−^) T cells. In a similar knockout mouse strain of survivin gene (*lck-Cre/survivin^fl/fl^*), for example, loss of survivin at this stage leads to a severe block in the DN to DP transition and more than 10-fold drop of the number of thymocytes [17], [18]. Unexpectedly, however, lck-FoxM1 mice only exhibit a two-fold reduction in the number of total thymocytes (Fig. 1A). The number of DN thymocytes remains similar and reduction starts in intermediated single positive (TCR^low^ CD8^+^CD4^−^) thymocytes (1.5 fold) followed by a 2-fold drop of DP thymocytes (Fig. 1A, 1B and data not shown). Positive selection (differentiation into CD4 or CD8 SP thymocytes) doesn't seem to be affected as the expression profiles of markers for positive selection, CD69 and HSA, are normal (data not shown). The CD25/CD44 staining profiles of DN thymocytes confirmed that loss of FoxM1 leads to accumulation of the DN3 thymocytes (Fig. 1B). Loss of FoxM1 in DP thymocytes and later T cell stages was confirmed using western blot analysis (see below). Analysis of the peripheral immune organs, spleen and lymph nodes also showed a similar modest drop of CD4 and CD8 mature T cells (Fig. 1C). We concluded that the absence of FoxM1 early during T cell development only modestly affects the transition of DN to DP T cells and has no effect in later stages of T cell differentiation.

### *CD4-Cre/FoxM1^fl/fl^* Mice Exhibit Normal T Cell Development but Defective Proliferation of Mature T Cells

**Figure 2:**
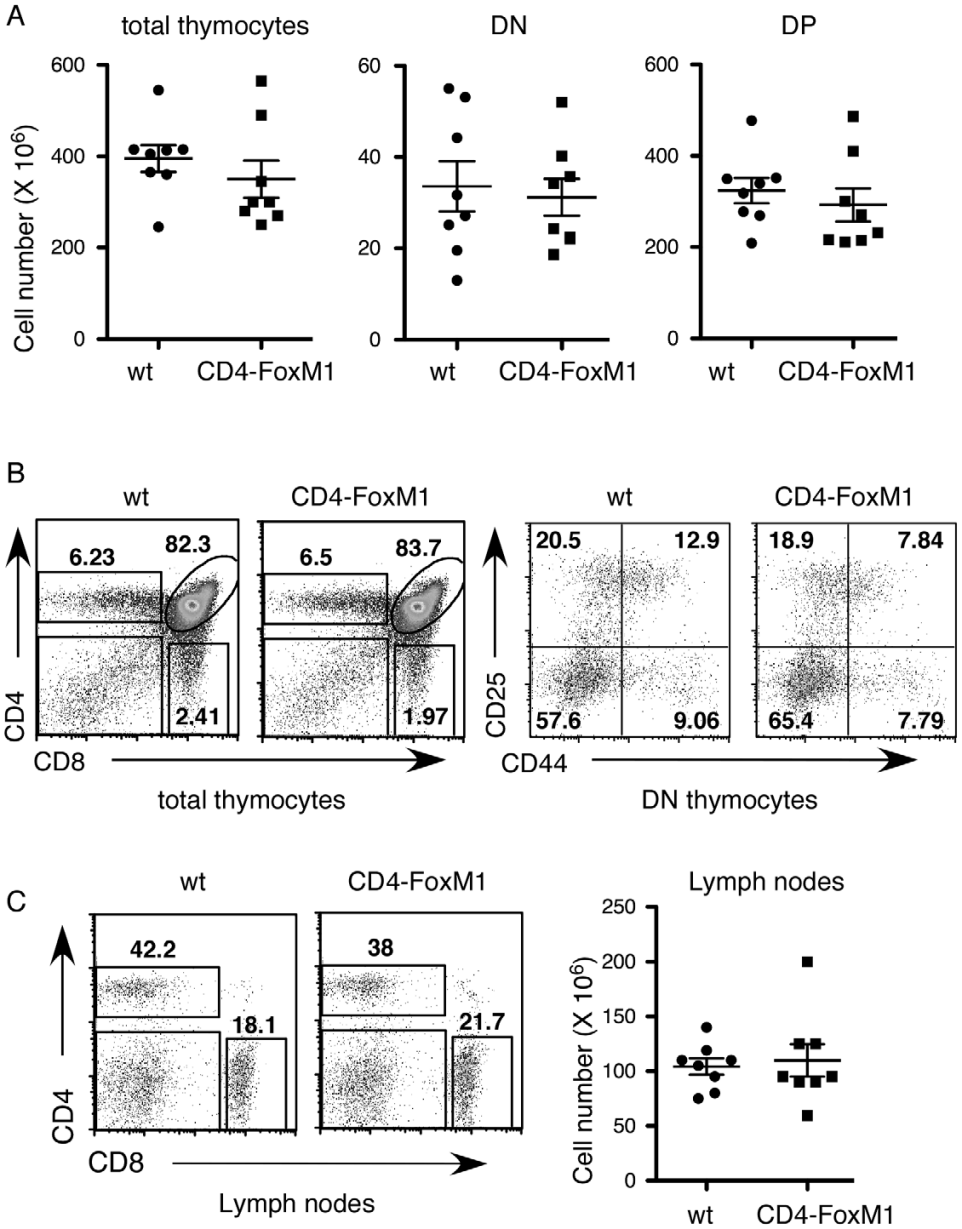
Characterization of CD4-FoxM1 mice. (A) The total number of thymocytes, double negative (DN), and double positive (DP) from each mouse are depicted as a box or a circle (*n* = 8). (B) Representative plots of CD4 *versus* CD8 profiles of total thymocytes and CD44 *versus* CD25 profiles of gated DN thymocytes are shown. (C) CD4 *versus* CD8 profiles and the total number of cells in lymph nodes are shown here. Right panel: Each box or circle represents one mouse (*n* = 8).

To bypass the requirement of FoxM1 in the proliferative stage of early T cell development, we bred the floxed *FoxM1* mice to the *CD4-Cre* transgenic mice [25] to generate *CD4-Cre/FoxM1^fl/fl^* mice (henceforth termed CD4-FoxM1). The Cre recombinase in *CD4-Cre* mice is not expressed until late stage of DN thymocytes [18] and thus FoxM1 should not be deleted until DP stage. In CD4-Cre survivin deficient mice, T cell development is normal in adult mice although the number of mature T cells drops due to defective homeostatic proliferation that takes place in the neonatal stage [18]. In CD4-FoxM1 mice, thymocyte cellularities and flow cytometric profiles are completely normal (Fig. 2), including the CD25/CD44 profile of their DN thymocytes. There is no difference of the percentages and total numbers of CD4/CD8 mature T cells in spleen and lymph nodes between CD4-FoxM1 mice and their wild-type controls (Fig. 2C). This later datum suggests that the neonatal homeostatic proliferation of mature T cells is normal in CD4-FoxM1 mice.

**Figure 3:**
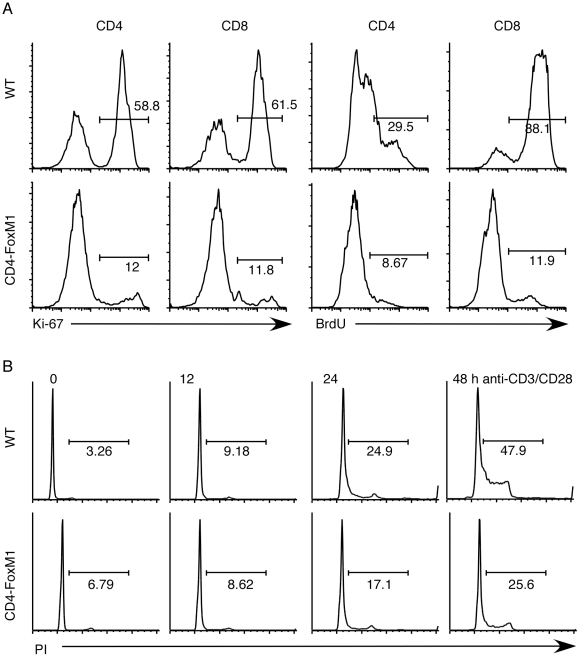
Reduced expression of FoxM1 affects proliferation of peripheral T cells. For both (A) and (B), purified peripheral T cells from CD4-FoxM1 mice or their wild-type littermate controls were stimulated with plate-bound anti-CD3/CD28 antibodies for 48 hr. (A) Left panel: non-G_0_ cells were measured by Ki-67 staining. The percentages of Ki67^+^ cells are as shown as the numbers in the figure. Right panel: Proliferation was measured using BrdU incorporation. The percentages of BrdU^+^ cells are as shown numbers in the figure. (B) Propidium iodide (PI) staining. The percentages of cells in both S and G_2_ phases were shown in the figure. The cell cycle analysis was done for the samples stimulated for 48 hr: wild-type (G_1_ = 34.9%, S = 57.8%, and G_2_/M = 5.9%) and CD4-FoxM1 (G_1_ = 56.3%, S = 33%, and G_2_/M = 5.82%). These experiments were repeated several times with similar results.

To see if FoxM1 is required for proliferation of mature T cells, we purified peripheral T cells from CD4-FoxM1 mice and subjected them to cross-linking by anti-CD3/CD28 antibodies. To measure the ability of these cells to enter the G_1_ cell cycle phase, we used Ki-67, a nuclear marker whose expression correlates with proliferation [27] and BrdU incorporation. As shown in figure 3A, while wild-type CD4 and CD8 T cells incorporated BrdU and expressed Ki-67, FoxM1-deficient T cells exhibited a dramatic reduction of both Ki-67 levels and BrdU incorporation. Propidium iodide staining further confirmed the reduction of cells in the S and G_2_/M phases of the cell cycle in FoxM1-deficient T cells (Fig. 3B) but no obvious block at the S to G_2_/M transition was seen. Interestingly, polyploidy cells were not observed. Thus, FoxM1 is a critical molecule for mature T cell during the early G_1_ to S transition.

### Loss of FoxM1 Leads to Loss of Cyclin B1 but Not Survivin in DP Thymocytes

**Figure 4:**
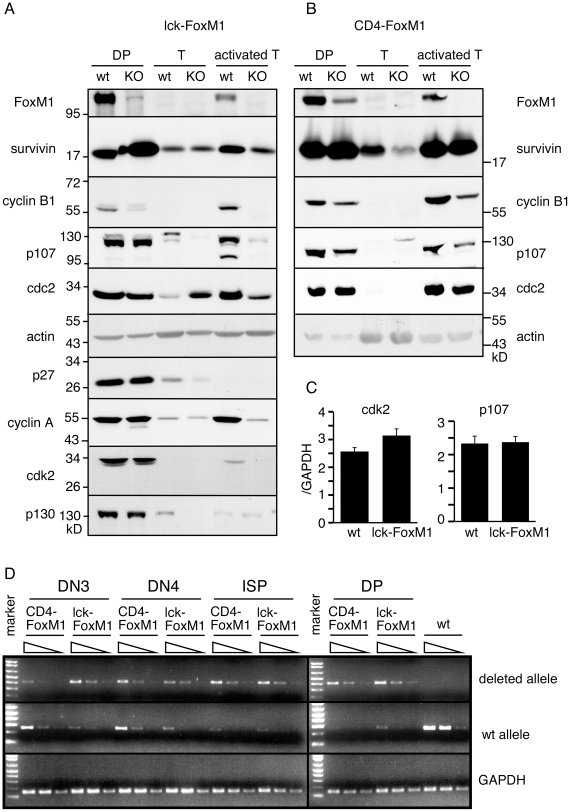
Western blot analysis of sorted T cell populations. Extracts from DP thymocytes, column-purified mature T cells (T) and anti-CD3/CD28 stimulated T cells (activated T) of (A) lck lck-FoxM1 or (B) CD4-FoxM1 were analyzed by western blot analysis using antibodies specific for FoxM1, survivin, cyclin B1, p107, cdc2, p27^kip1^, cyclin A, cdk2, p130 and anti-actin for loading control. Western blot analysis of lck-FoxM1 cells using antibodies specific for p27, cyclin A, cdk2 and p130 was also carried out. (C) Quantitative real-time PCR of cdk2 and p107 using sorted DP thymocytes of lck-FoxM1 mice and their wild-type littermate controls. These experiments were repeated several times with similar results. (D) Semi-quantitative PCRs of the FoxM1 deleted and wild-type alleles using primers as described in [36].

As a master regulator of cell cycle genes, loss of FoxM1 was expected to lead to a wide spread dys-regulation of cell cycle proteins. To see if this is the case, we isolated cell extracts from sorted DP thymocytes, purified naïve T cells and activated mature T cells. Western blot analysis was then carried out using antibodies specific for each individual cell cycle protein. As we reported previously, DP thymocytes express many cell cycle proteins [21]. Expression of these proteins is extinguished following positive selection but is re-activated when mature T cells are stimulated to undergo proliferation. In DP cells of lck-FoxM1 mice, only residual FoxM1 protein was detected (Fig. 4A). In contrast, the level of FoxM1 was reduced but not eliminated in DP cells of CD4-FoxM1 mice (Fig. 4B), presumably due to the long half-life of the FoxM1 protein. Activated T cells from both lines of mice had undetectable levels of FoxM1. The difference in FoxM1 protein levels between these two strains of mice is likely due to the differential kinetics of FoxM1 deletion during T cell development. Consistent with the earlier reports [18], [25], semi-quantitative PCR analysis of the deleted and wild-type FoxM1 alleles from lck- and CD4-FoxM1 mice showed higher levels of FoxM1 deletion in DN3 and DN4 thymocyte populations of lck-FoxM1 mice (Fig. 4D). Surprisingly, survivin levels were largely unchanged in DP cells and activated T cells of both lck-FoxM1 and CD4-FoxM1 mice (Fig. 4A, B). This is different to what others have found in FoxM1 knockdown cell lines [28]. A small reduction of survivin level could be consistently seen in naïve mature T cells of CD4-FoxM1 mice. However, since FoxM1 is normally not expressed in naïve T cells, this reduction is most likely due to an indirect effect of FoxM1 deficiency. As expected, cyclin B1 level was severely impacted by loss of FoxM1 in DP and activated T cells of lck-FoxM1 mice. It was reduced in DP and activated T cells from CD4-FoxM1 mice. No other cell cycle proteins examined so far were affected in DP thymocytes of lck-FoxM1 or CD4-FoxM1 mice. These include the Rb family members p107 and p130, cdc2, cdk2, cyclin A and the cell cycle inhibitor p27 (Fig. 4A, B). Quantitative RT-PCR of p107, CDK2, cyclin E and p130 showed that transcripts of these genes didn't go down in FoxM1-deficient DP cells from lck-FoxM1 mice (Fig. 4C and data not shown). In contrast to thymocytes, TCR stimulated FoxM1-deficient mature T cells had lower or very little expression of p107, cdc2, cyclin A and cdk2 compared to their wild-type controls. FoxM1-deficient naive T cells also expressed lower levels of p27 and p130 (Fig. 4A). We concluded that FoxM1 is a master regulator of cell cycle proteins in mature T cells but not in DP thymocytes.

### Normal Levels of Apoptosis in FoxM1 Deficient DP Thymocytes

**Figure 5:**
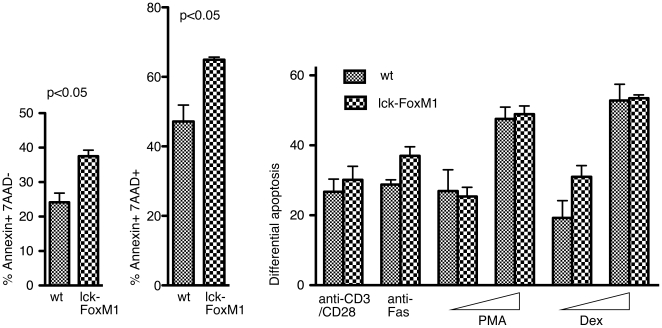
Lack of FoxM1 expression increased apoptosis of DP thymocytes in culture. Left panel: apoptosis of DP thymocytes (Annexin V^+^ 7ADD^−^) culture *in vitro* for 24 hr. Middle panel: total number of cell death as measured by Annexin V^+^ 7ADD^+^ of DP thymocytes culture *in vitro* for 24 hr. Right panel: DP thymocytes from Lck-FoxM1 mice or their wild-type littermate controls were stimulated with anti-CD3/CD28, anti-Fas antibody, PMA phorbol ester (PMA) or dexamethasone (Dex). The extent of apoptosis (% Annexin V positive, 7-AAD negative cells) was measured 24 hours later.

The cell cycle status might affect the sensitivity to apoptosis [29]. Expression of the cell cycle proteins in DP cells might thus result in their inherent sensitivity to cell death. To see if loss of FoxM1 might affect apoptosis of DP cells, we measured apoptosis of lck-FoxM1 DP thymocytes when cultured *in vitro* and when stimulated with different apoptotic-inducing signals. Cultured FoxM1-deficient DP thymocytes were slightly more sensitive to apoptosis and cell death (Fig. 5). However, no changes in differential apoptosis were observed when apoptotic stimuli (anti-CD3/CD28, anti-Fas antibodies, phorbol ester PMA or dexamethasone) were added. It is not clear how loss of FoxM1 contributes to increased sensitivity of cell death in DP cells. Interestingly, siomycin A and thiostrepton, two chemical inhibitors of FoxM1, can induce cell death in transformed cells [30], [31].

## Discussion

FoxM1, a member of the forkhead box family of transcription factors, is a known master regulator of cell cycle-specific genes. In cell lines and several primary tissues, FoxM1 was shown to directly regulate many cell cycle-specific genes, including the G_1_/S specific genes (cyclin D1, Skp2 and Cks1), the S phase-specific gene c-myc, the G_2_/M genes (cdc25B and cyclin B1) and those that are involved in mitosis (aurora, survivin and CENP) [15]. During T cell development, FoxM1 is expressed at a high level in DP thymocytes and proliferating DN thymocytes [19], [20]. The expression level of FoxM1 is down-regulated as DP cells differentiate into CD4 or CD8 SP T cells. FoxM1 is undetectable in naïve T cells but is re-expressed when mature T cells are activated to proliferate through their TCR. We are interested in understanding why DP thymocytes are not proliferating but they express many cell cycle proteins, including FoxM1 [21], [22]. DP thymocytes are exquisitely sensitive to apoptosis. We hypothesize that the sensitivity to apoptosis is due to the widespread expression of cell cycle proteins. We'd thus like to see if loss of FoxM1 might lead to global down-regulation of cell cycle activities in DP thymocytes and resistance to apoptosis.

We have generated and analyzed two lines of T-cell specific FoxM1 deficient mice. In lck-FoxM1 mice, the FoxM1 exons (exon 4–7) were deleted at the early DN stage and this occured at a higher efficiency than CD4-FoxM1 mice. Reduction of FoxM1 protein is fairly complete by the time thymocytes differentiate into CD4^+^CD8^+^ DP cells. Loss of FoxM1 at the early stage in lck-FoxM1 mice affects DN to DP transition, resulting in a two-fold drop of the DP thymocytes followed by a similar reduction of SP thymocytes and mature T cells. This modest phenotype is different from that of *lck-Cre/survivin^fl/fl^* mice, which suffer from an almost complete block of DN to DP development [18]. Residual survivin protein could also be seen in DP thymocytes of *lck-Cre/survivin^fl/fl^* mice. The difference in the requirement of survivin versus FoxM1 in early T cell development could be due to an unknown regulator that compensates for the loss of FoxM1 in early T cells. Alternatively, FoxM1 protein might be more stable than survivin and thus there is just enough protein for DN to undergo proliferation despite deletion of their FoxM1 alleles.

Surprisingly, loss of FoxM1 has no effect on most of the cell cycle proteins expressed in DP thymocytes. DP thymocytes are an unusual group of cells. They do not proliferate but yet they express all the cell cycle proteins examined to date [21], [32]. These include FoxM1, survivin, cyclin B1, cyclin A, cdc2, cdk2, cyclin E, p21, p27 and the Rb family members Rb, p107 and p130. Intracellular staining studies with antibodies against a few of these proteins showed that their expression is not restricted to a subgroup of DP cells but in all DP cells [21]. As DP cells mature into SP thymocytes, expression of these proteins is extinguished. Down-regulation can also be seen at the transcription levels [22]. Given the role of FoxM1 as a master cell cycle regulator in other cell types [23], [28], [33], including mature T cells (this report), it is surprising to see that cyclin B1 is the only protein whose expression is being affected in FoxM1-deficient DP thymocytes. No changes were found for p107, cdc2, p21, cyclin A, cdk2, p130, p27 or survivin levels. As cyclin B1 is affected, it is unlikely that the residual FoxM1 protein can be responsible for the normal expression levels of these other cell cycle genes. One possibility is the presence of yet another master regulator that compensates for the loss of FoxM1.

The lack of effect of FoxM1 deficiency on survivin levels is surprising. Survivin has been shown to be a direct target of FoxM1 in cell lines and in primary colon cells [28], [30], [34]. Even in activated mature T cells where many of the cell cycle proteins are being affected by FoxM1, survivin protein could be detected at a significant level. It is possible that transcriptional regulation of survivin by FoxM1 is cell type dependent. Further research will be necessary to identify the upstream regulator of survivin in T cells and to understand the molecular mechanisms that regulate cell cycle levels in DP thymocytes.

## Materials and Methods

### Ethics Statement

All animals were handled in strict accordance with good animal practice as defined by the relevant national and/or local animal welfare bodies, and all animal work was approved by the campus Animal Care and Use Committee.

### FoxM1 Knockout Mice

T-cell conditional FoxM1 knockout mice were generated by crossing *FoxM1^flox/flox^* mice to *Lck-Cre* or *CD4-Cre* transgenic mice, which express Cre recombinase at different DN stages. For genotyping *Cre/FoxM1^flox/flox^* mice, two pairs of primers were used for PCR: Cre 5′ (CCAGCTAAACATGCTTCATCGTC) and Cre 3′ (CCTGATCCTGGCAATTTCGG); FoxM1 5′ (TAGGAGATACACTGTTATAT) and FoxM1 3′ (TGTGGGAAAATGCTTACAAAAG).

### Flow Cytometric Analysis

Cells were prepared from lymphoid organs of littermates. After red blood cell lysis, they were stained with the indicated antibodies. Anti-CD4, anti-CD8, anti-TCRβ, anti-CD44, and anti-CD25 antibodies were purchased from BD Pharmingen. Intracellular staining was performed as described previously [35]. Briefly, formaldehyde was added directly to culture medium to a final concentration of 2% and incubated for 10 min at room temperature. The cells were pelleted, resuspended in ice-cold methanol, and incubated for 15–30 minutes on ice. The cells were then washed three times with staining buffer (0.5% BSA in PBS) and stained with antibodies of interest. Fresh isolated thymocytes and peripheral T cells were fixed and stained with anti-Ki-67 antibody. Column-purified peripheral T cells were stimulated with plate-bound anti-CD3 (2 µg/mL) and anti-CD28 (2 µg/mL) for 48 hours, fixed and then stained with anti-Ki-67 antibody. Anti-Ki-67 antibody was purchased from BD Bioscience. BrdU incorporation was performed as following: column-purified peripheral T cells were stimulated with plate-bound anti-CD3 (2 µg/mL) and anti-CD28 (2 µg/mL) for 24 hours and 10 µM of BrdU was added into cell culture medium for another 16 hours. The BrdU staining was measured after cells were fixed. The anti-BrdU antibody was purchased from Caltag. PI staining was performed by re-suspending cells in PBS containing 200 µg/ml RNase, 50 µg/ml propidium iodide, 0.1% sodium citrate, and 0.1% (v/v) Triton X-100.

### Western Blotting

Cell lysates were prepared from sorted DP thymocytes, CD4^+^CD8^−^ SP thymocytes, CD4^+^ peripheral T cells or CD8^+^ peripheral T cells. The following antibodies used in this study: anti-FoxM1, anti-cdc2, anti-cdk2, anti-cyclin A, anti-p130, anti-p27, anti-p107, anti-survivin and anti-cyclin B1 antibodies were purchased from Santa Cruz Biotechnology; and anti-actin antibody was purchased from Abcam.

### Apoptosis Assay

Thymocytes was isolated and stimulated with plate-bound anti-CD3 (2 µg/mL) and anti-CD28 (2 µg/mL), anti-Fas (2 µg/mL), PMA (0.6, 1.2 ng/mL) or Dexamethasone (4.5, 9.0 nM). After the indicated time, cells were collected and stained with cell surface markers, CD4 and CD8, as well as Annexin V (BD Biosciences) and 7-AAD for flow cytometric analysis.

### Quantitative Real-Time RT-PCR

Total RNAs were extracted from sorted DP thymocytes. The primer sequences used in RT-PCR are: CDK2 forward (5′-GCACGATCCATACCCTCTGT) and reverse (5′-CCCATTTTGGTCTGCTCAAT), p107 forward (5′-GGAGATTGGAACACCTCGAA) and reverse (5′-ATACCGCCGTCCAGTAAGTG), as well as GAPDH forward (5′-ACCCAGAAGACTGTGGATGG) and reverse (5′-GGATGCAGGGATGATGTTCT).

Semi-quantitative PCR. Genomic DNAs were extracted from sorted DN3, DN4, ISP (intermediate single positive) and DP thymocytes of wild-type, CD4-FoxM1 or lck-FoxM1 mice. The primer sequences used in PCR for the FoxM1 deleted allele are forward (5′-TGGCTTCCCAGCAGTACAAATC) and reverse (5′- TCTCGCTCAATTCCAAGACCAG); the primers for the FoxM1 wild-type allele are forward (5′-TGGCTTCCCAGCAGTACAAATC) and reverse (5′-TGCTTACAAAAGACACACTTGGACG); and GAPDH forward (5′-TGGCAAAGTGGAGATTGTTGCC) and reverse (5′-AAGATGGTGATGGGCTTCCCG). The amounts of genomic DNA used for semi-quantitative PCR were 2 mg/L, 0.4 mg/L, and 0.08 mg/L.
